# Single-Parent Expression of Anti-sense RNA Contributes to Transcriptome Complementation in Maize Hybrid

**DOI:** 10.3389/fpls.2020.577274

**Published:** 2020-12-03

**Authors:** Xiangbo Zhang, Yongwen Qi

**Affiliations:** Guangdong Sugarcane Genetic Improvement Engineering Center, Institute of Bioengineering, Guangdong Academy of Sciences, Guangzhou, China

**Keywords:** maize, antisense, hybrid, single-parent expression, epigenetic modification

## Abstract

Anti-sense transcription is increasingly being recognized as an important regulator of gene expression. But the transcriptome complementation of anti-sense RNA in hybrid relative to their inbred parents was largely unknown. In this study, we profiled strand-specific RNA sequencing (RNA-seq) in a maize hybrid and its inbred parents (B73 and Mo17) in two tissues. More anti-sense transcripts were present in the hybrid compared with the parental lines. We detected 293 and 242 single-parent expression of anti-sense (SPEA) transcripts in maize immature ear and leaf tissues, respectively. There was little overlap of the SPEA transcripts between the two maize tissues. These results suggested that SPEA is a general mechanism that drives extensive complementation in maize hybrids. More importantly, extremely high-level expression of anti-sense transcripts was associated with low-level expression of the cognate sense transcript by reducing the level of histone H3 lysine 36 methylation (H3K36me3). In summary, these SPEA transcripts increased our knowledge about the transcriptomic complementation in hybrid.

## Introduction

Heterosis is defined as the super performance of a primary hybrid (F_1_) compared with its homologous parental lines (Flint-Garcia et al., 2009; Birchler et al., 2010) with respect to characters related to vigor, such as disease resistance, plant architecture, and crop yield. At present, many hypotheses related to the mechanisms governing heterosis have been suggested at the level of the transcriptome, such as the possible role of additive (Guo et al., 2006; Stupar and Springer, 2006; Swanson-Wagner et al., 2006; Meyer et al., 2007; Springer and Stupar, 2007; Stupar et al., 2008; Thiemann et al., 2010, 2014) or non-additive gene expression (Auger et al., 2005; Użarowska et al., 2007; Hoecker et al., 2008; Pea et al., 2008; Swanson-Wagner et al., 2009; Jahnke et al., 2010; Paschold et al., 2010; Baldauf et al., 2016).

Recently, single-parent expression (SPE) has been shown to be an important mechanism contributing to heterosis (Paschold et al., 2012, 2014; Baldauf et al., 2016, 2018; Marcon et al., 2017). SPE refers to genes that are active in one parent and inactive in the other parent while also being active in the F_1_ hybrid. These studies reported that transcriptome complementation, which is the complementation of deleterious parental alleles by superior alleles of the second parent, contributes to maize heterosis in hybrids relative to its inbred parental lines. These SPE transcripts are enriched in non-syntenic genes (Baldauf et al., 2018). Non-syntenic genes evolved after the separation of maize and sorghum, which might explain the important role of non-syntenic genes in maize heterosis. However, the regulatory mechanisms that control SPE transcripts are presently unknown.

Anti-sense RNA is a class of non-coding RNAs that are transcribed from the strand that is opposite to the sense transcripts of protein-coding genes, and anti-sense RNA plays an important role in regulating gene expression through various mechanisms. In *Arabidopsis*, anti-sense transcripts are produced from ∼30% of all protein coding genes (Yamada et al., 2003; Ietswaart et al., 2012). In human tissues, anti-sense transcripts are co-expressed or are expressed in an inverse correlation with sense transcripts (Chen et al., 2005; Xu et al., 2011). Anti-sense transcript can control gene expression by affecting cytosine the methylation level or histone modification (Pelechano and Steinmetz, 2013). For example, *ANRIL* (anti-sense ncRNA) in the *INK4* locus, which is increasingly expressed in prostate cancer, affects the specific repression of the tumor suppressor locus, which encodes p15 (also known as INK4B), p14 (also known as ARF), and p16 (also known as INK4A). Interestingly, gene body is associated with increasing expression level of anti-sense transcripts by reducing histone H3 lysine 36 methylation (H3K36me3) modification (Murray et al., 2015).

Transcriptome complementation (sense transcript) is a common phenotype in hybrid to its parental lines. However, it is also important to detect SPE of anti-sense (SPEA) transcript, which contributes to transcriptome complementation in hybrid. In this study, we performed strand-specific RNA sequencing (RNA-seq) in three maize genotypes (B73, Mo17, and their F_1_ hybrid) and two tissues (immature ear and leaves). The purpose of this study was to (1) discover the pattern of SPEA; (2) examine the correlation between SPEA (the expression levels of anti-sense transcripts) and SPE (the expression level of sense transcript), and (3) examine whether H3K36me3 participates in the regulation module of SPEA and SPE.

## Results

### Profiling Anti-sense Transcript in Maize

Single-parent expression is a general mechanism to explain maize heterosis with more genes being expressed in a hybrid than in its parents. Anti-sense transcripts participated in the regulation of their cognate sense transcripts via various pathways (Pelechano and Steinmetz, 2013). Therefore, it is of interest to study the dynamic expression pattern of anti-sense transcripts in parental lines and their progeny. In this study, the generation of strand-specific transcriptome data from immature ears and 14-day-old leaves in B73, Mo17, and their hybrid progeny provided an opportunity to study the role of anti-sense RNA on transcriptome complementation. A total of 18 RNA samples (three genotype × three biological replicates × two tissues) were prepared for strand-specific RNA-seq on an illumine platform, which generated 150-bp paired-end reads. After low-quality reads were filtered out and the barcode sequences from reads were trimmed, approximately 113 gigabyte (Gb) of sequencing data was obtained (Supplementary Table 1). Between 88 and 97% of all reads are mapped to the maize genome. The unique mapping efficiency ranged from 75 to 79% (Supplementary Table 1). The Pearson correlation coefficients (PCCs) for the three biological replicates ranged from 0.98 to 1.00 (Supplementary Figure 1). To estimate the specificity of each library, the directions of the reads were mapped to 3,962 genes, with no annotated transcripts within 3 kb. The strand specificity of the 18 libraries varied from 99.1 to 99.5%, which showed the high level of strandness for all of the sequencing libraries. These results suggested that the strand-specific RNA-seq data were of high quality.

To systematically analyze the expression level of anti-sense and sense transcripts in B73, Mo17, and their hybrid, sense transcripts were obtained based on the longest annotated transcripts for each gene (B73 RefGen_V4), and anti-sense transcripts were obtained by assembling reads (opposite direction to the sense transcript) using StringTie software (Pertea et al., 2015). Both of the sense and anti-sense transcripts that originated from the same gene were defined as natural anti-sense transcript (NAT) pairs. To analyze the correlation between replicates determined by anti-sense profiling, PCCs were calculated with the expression value of anti-sense transcript (Supplementary Figure 2). Overall, PCCs ranged from 0.95 to 0.99, which were lower than PCCs calculated by gene expression level (Supplementary Figure 1) (mixture of sense and anti-sense transcripts). This was not surprising because the relatively low abundance of anti-sense transcript might weaken the reproducibility of replicate samples.

**FIGURE 1:**
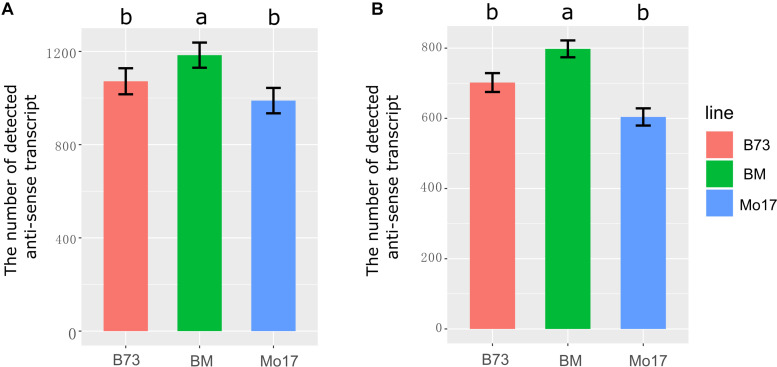
The number of detected anti-sense RNA in immature ear and leaves in B73, Mo17, and their hybrid. **(A)** Immature ear. **(B)** Leaves. Different letters above the histogram indicate significant differences (*P* < 0.05, Duncan multiple test). The ylab represents the number of detected anti-sense transcripts.

The number of expressed genes in the hybrid was higher than in the two parental lines, which contributed to heterosis in the hybrid (Paschold et al., 2012; Baldauf et al., 2016, 2018). Therefore, it was necessary to answer the question that whether more anti-sense transcripts were activated in the hybrid. In total, 1,613 NAT pairs were identified in at least one sample. Significantly, the number of detected anti-sense transcripts was higher in the F_1_ than in its parents in immature ears and leaves (Figures 1A,B, *P* < 0.05). We detected 1,072, 989, and 1,184 anti-sense transcripts in B73, Mo17, and the F_1_, respectively, in immature ears (Figure 1A). In leaves, 702, 604, and 798 anti-sense transcripts were detected in B73, Mo17, and their hybrid, respectively (Figure 1B). These results suggested that transcriptome complementation of anti-sense transcript might be a general phenomenon in hybrid relative to its parental lines.

### Kinship Relationships Among a Diverse Panel of Genotypes Detected by Anti-sense RNA Profiling

**FIGURE 2:**
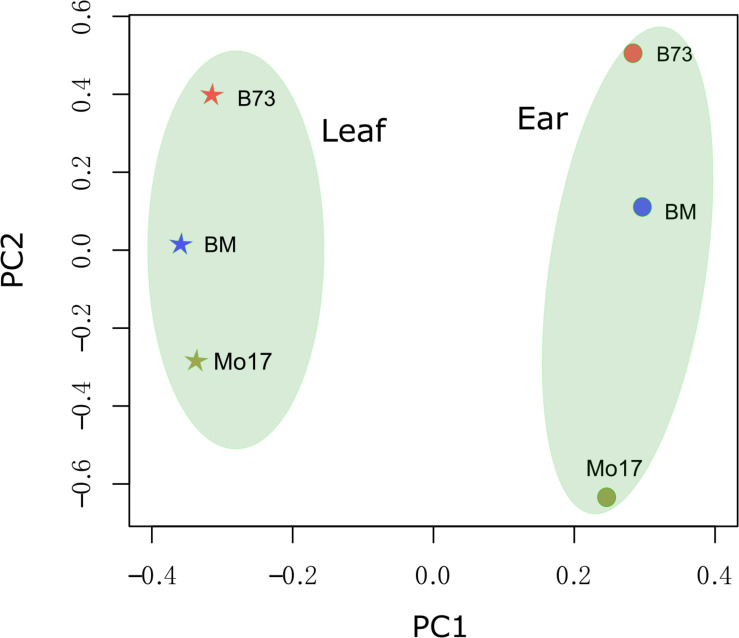
Principal component analysis (PCA) performance for strand-specifically sequencing samples. The symbol “BM” represents the hybrid between B73 and Mo17 (B73 × Mo17). The lines B73, Mo17, and BM are represented by orange, blue, and yellow, respectively. The immature ear and leaf tissues are represented by a circle and a pentagram, respectively.

Diverse genotypes and developmental stage tissues could be separated by transcriptome data (mixture of sense and anti-sense transcript) (Baldauf et al., 2016, 2018; Zhou et al., 2019). Anti-sense RNA was considered to be transcriptional “noise” when it was first discovered (Pelechano and Steinmetz, 2013). It was interesting that anti-sense RNA could be used to distinguish different genotypes and tissues. The expression level of anti-sense transcript was used to perform principal component analysis (PCA) (Figure 2). Principal Component 1 (PC1) and Principal Component 2 (PC2) could explain 36.1 and 16.4% of the variance, respectively. PC1 could distinguish maize tissues (immature ear from leaves). PC2 could distinguish the three genotypes (B73, Mo17, and the F_1_), where the F_1_ was located in a position between the locations of its parents.

### Single-Parent Expression Complementation of Anti-sense Transcript Varied Among Maize Tissues

**FIGURE 3:**
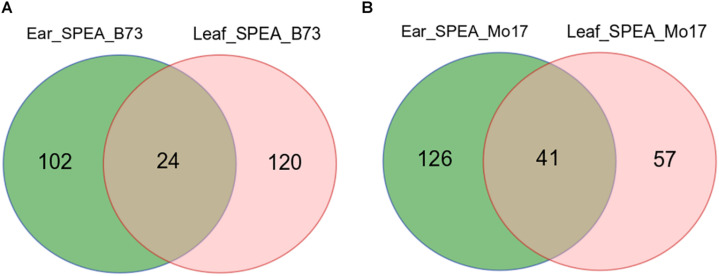
Venn plot of detected single-parent expression of anti-sense (SPEA). **(A)** SPEA_B (single-parent expression of anti-sense in B73) in two tissues. **(B)** SPEA_M (single-parent expression of anti-sense in Mo17) in two tissues.

Single-parent expression was determined by comparing the extremely expressed genes between the F_1_ and its parents. SPEA was detected by comparing the expression levels of anti-sense transcripts in B73 and Mo17. SPEA RNA was defined as transcript that expressed in one parent [fragments per kilobase per million (FPKM) ≥ 0.2] but not expressed in the other parent (FPKM ≤ 0.05). By this criterion, 293 SPEA genes were identified in the immature ear tissue. Among them, 126 genes showed higher expression in B73 (SPEA_B), and 167 genes showed higher expression in Mo17 (SPEA_M) (Figure 3A). Also, 242 SPEA genes were detected in maize leaf tissue. Of these, 144 genes showed higher expression in B73 (SPEA_B), and 98 genes showed higher expression in Mo17 (SPEA_M) (Figure 3B). Interestingly, only 24 SPEA were detected in both B73 tissues (Figure 3A), while 41 SPEA was detected in both Mo17 tissues (Figure 3B). This result suggested that SPEA was differentiated between the two tissues.

To obtain an estimate of the proportion of genes that display SPEA because of genes that are not present in one of the parental inbred lines, the present and absence variation (PAV) genes that are present in B73 and absented in Mo17 were obtained from previously published study (Sun et al., 2018). For SPEA_B detected in the immature ear, only ∼5% of the genes were due to PAV (by permutation test; *P* > 0.05). For SPEA_B detected in leaves, only 7% of the genes were due to PAV (by permutation test; *P* > 0.05). We were not able to detect genes that were physically presented in Mo17 but not in B73, because the transcriptome data were mapped to the maize B73 reference genome. These results suggested that most genes displaying SPEA patterns were physically presented in the genome but were only transcriptionally active in one genotype and inactive in the other genotype.

### An Antagonistic Regulation Relationship Between Anti-sense and Its Cognate Sense Transcripts

**FIGURE 4:**
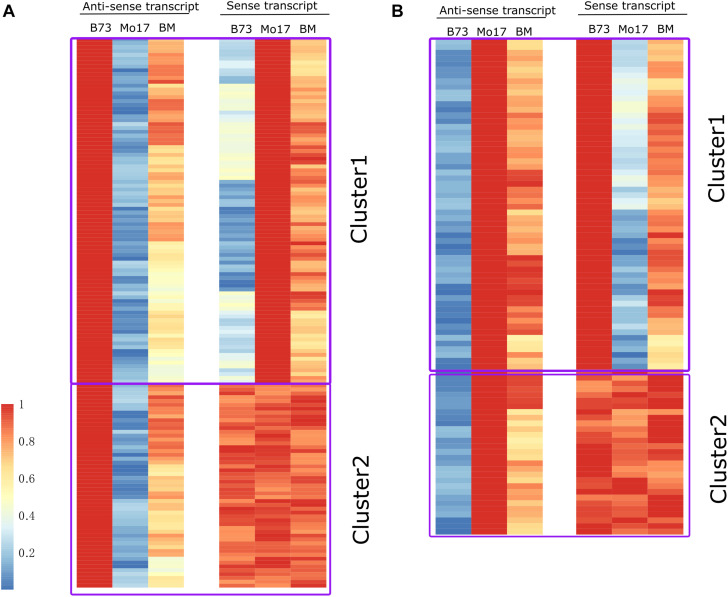
Dynamic pattern of single-parent expression of anti-sense (SPEA) and its cognate sense RNA identified in leaf tissue in three genotypes (B73, Mo17, and BM). **(A)** Expression heatmap of SPEA_B and its cognate sense transcript in B73, Mo17, and BM. **(B)** Expression heatmap of SPEA_M and its cognate sense transcript in B73, Mo17, and BM. The fragments per kilobase per million (FPKM) value for anti-sense and sense transcript was normalized by their maximum observed FPKM. The red and blue colors represent the relatively high and low expression level. For SPEA_B in cluster 1 **(A)**, Pearson correlation coefficient (PCC) between anti-sense and sense transcript was -0.86 (*P* < 1 × 10^–21^). For SPEA_M in cluster 1 **(B)**, PCC between anti-sense and sense transcript was -0.89 (*P* < 1 × 10^–18^).

We detected 242 and 293 SPEA in leaf and immature tissues, respectively (Figure 3). These SPEA were also expressed in F_1_ (Figures 4A,B and Supplementary Figures 3A,C). To determine whether these SPEA could regulate the expression levels of their cognate sense mRNAs, we further analyzed the expression pattern of its cognate sense transcripts in B73, Mo17, and their hybrid.

(1) For leaf tissue, among the total 144 SPEA_B, their cognate sense transcripts could be divided into two clusters (cluster 1 and cluster 2) based on the expression pattern between B73 and Mo17 (Figure 4A). Sense transcripts in cluster 1 (70.8%) showed the opposite expression pattern compared with their cognate anti-sense transcript (Figure 4B). Sense transcripts in cluster 2 (28.2%) did not differentiate expression between B73 and Mo17 (Figure 4A). Also, the corresponding sense transcript of SPEA_M could also be divided into two clusters, where sense transcripts in cluster 1 (71.43%) showed an opposite expression trend as compared with their anti-sense transcripts (Figure 4B). Furthermore, PCCs between sense and anti-sense transcripts was -0.55 (*P* < 1 × 10^–8^) and -0.52 (*P* < 1 × 10^–11^) for gene set of SPEA_B and SPEA_M, respectively (Supplementary Figure 4).
(2) For immature tissue, among the total 126 SPEA_B, their cognate sense transcripts could be divided into two clusters (cluster 1 and cluster 2) based on the expression pattern between B73 and Mo17 (Supplementary Figure 3B). Sense transcripts in cluster 1 (73.0%) showed the opposite expression pattern compared with their cognate anti-sense transcripts (Supplementary Figure 3B), while sense transcript in cluster 2 (27.0%) did not show differentiated expression between B73 and Mo17 (Supplementary Figure 3B). We further analyzed SPEA_M genes (Supplementary Figures 3C,D). Also, the corresponding sense transcript of SPEA_M could also be divided into two clusters, where cluster 1 (76%) showed an opposite expression trend as compared with their anti-sense transcript (Supplementary Figure 3D).

Altogether, the negative correlation between sense and anti-sense transcript was observed for gene set of SPEA_B and SPEA_M (Figure 4 and Supplementary Figures 3, 4).

### H3K36me3 Participated in the Antagonistic Regulation Module of Sense and Anti-sense RNA

**FIGURE 5:**
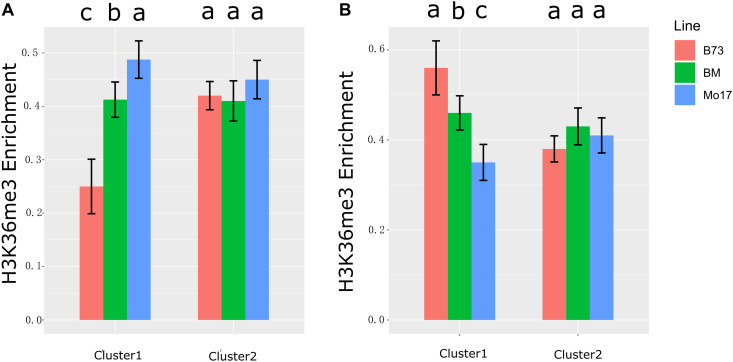
H3K36me3 enrichment in gene body. **(A)** H3K36me3 enrichment of genes in cluster 1 and cluster 2 of Figure 4A (SPEA_B). **(B)** H3K36me3 enrichment of genes in cluster 1 and cluster 2 of Figure 4B (SPEA_M). The different colors of histogram represent three maize lines (B73, Mo17, and BM). The *y*-axis represents the normalized H3K36me3 signal of gene body (see section “Materials and Methods” for details). Different letters above the histogram indicate significant differences (*P* < 0.05, Duncan multiple test).

It has been reported that anti-sense transcription was associated with reducing H3K36me3 level (Murray et al., 2015). In this study, we first discovered anti-sense RNA (for SPEA) inhibited its cognate sense transcript expression (Figure 4 and Supplementary Figures 3, 4). To further associate H3K36me3 modification with the expression of anti-sense and sense transcripts, H3K36me3 profiling data in B73, Mo17, and their hybrid in leaf tissue were obtained from public paper (He et al., 2013). We re-analyzed H3K36me3 data based on B73 reference genome (Version 4, see section “Materials and Methods” for details). We integrated H3K36me3 data with the expression level of NAT pairs identified in maize leaf tissue. For SPEA_B in cluster 1 (the expression level of sense and anti-sense transcripts was negatively correlated) (Figure 4A), H3K36me3 level was downregulated in B73 than Mo17 (*P* < 0.05, B73 < hybrid < Mo17) (Figure 5A). For NAT pairs in cluster 2 (the expression level of anti-sense transcript was not associated with its cognate sense transcript), H3K36me3 modification level was non-differentiated among B73, Mo17, and their hybrid (*P* > 0.05, Figure 5A). This result suggested that ∼71.8% SPEA_B (Figure 4A, cluster 1) was negatively associated with the expression of sense transcript and modification level of H3K36me3. The same result was also found in SPEA_M (Figure 5B). Altogether, these results suggested that anti-sense transcript (SPEA) might inhibit sense transcript expression by reducing modification level of H3K36me3, which enriched our knowledge of the mechanism about transcriptome complementation.

### Experimental Validation of Natural Anti-sense Transcript Pairs

Four genes were randomly selected for experimental validation. The primer sequences are listed in Supplementary Table 3. The expression level of these four genes was quantified by quantitative real-time PCR (qPCR) in immature ear (Zm00001d008203 and Zm00001d033303) and leaves (Zm00001d045478 and Zm00001d023311) in maize B73 and Mo17 inbred lines. All four sense and anti-sense transcript pairs were successfully detected, and changes in expression were consistent with strand-specific transcriptome sequencing (Supplementary Figure 5).

## Discussion

Anti-sense transcription, which was initially considered to be transcriptional noise, is increasingly being recognized as an important regulator of gene expression. Anti-sense RNA has been found to be widespread in humans (Guil and Esteller, 2012), bacteria (Georg and Hess, 2011; Sesto et al., 2013), and plants (Ietswaart et al., 2012). In this study, a maize F_1_ hybrid and its parental inbreds (B73 and Mo17) were clearly distinguished from one another by anti-sense RNA profiling (Figure 2). This result was consistent with previous studies about transcript profiling in various maize tissues and genotypes (Baldauf et al., 2018; Zhou et al., 2019). The results of our study suggested that the expression of anti-sense transcript differed among genotypes and that anti-sense transcript was an important factor that might be involved in the regulation of plant growth.

Single-parent expression is a general mechanism that drives extensive complementation in maize (Baldauf et al., 2018; Zhou et al., 2019) and rice hybrids (Shao et al., 2019). The super performance of F_1_ hybrid could be explained by the increasing number of expressed genes compared with the parental lines. At present, the regulatory mechanisms controlling gene expression in the hybrid and its parents were largely unknown. Anti-sense RNA was a class of non-coding RNA that regulated annotated transcripts in various ways (Pelechano and Steinmetz, 2013). Therefore, we speculated that SPEA RNA might be involved in regulating sense RNA expression. In this study, we identified 242 SPEA transcripts in leaves (Figure 3). Approximately 71.07% of the sense transcripts showed an opposite expression trend compared with the anti-sense RNA in B73 and Mo17 (Figure 4). This result suggested that anti-sense RNA might contribute to transcriptome complementation by regulating the expression of sense RNA.

In total, we detected 293 and 242 SPEA in maize immature ear and leaves, and the overlap of SPEA_B or SPEA_M between the two tissues was less (Figure 3). Previous studies also reported less overlap of SPE among different tissues by transcript profiling of RNA (mixture sense and anti-sense) (Baldauf et al., 2018; Shao et al., 2019). This result showed that transcriptome complementation of both anti-sense and sense transcript was differentiated among tissues.

In this study, we observed that sense transcript was negatively regulated by anti-sense transcript (Figure 4). But the regulated mechanism was not clear. A previous study took a genome-wide approach to assess various chromatin features associated with nascent anti-sense transcription. Gene body was associated with increasing expression of anti-sense by reducing H3K36me3 modification (Murray et al., 2015). By using mutants in which the level of anti-sense transcription was reduced at *GAL1*, or altered genome-wide, it was found that anti-sense transcription was associated with reducing H3K36me3 modification (Murray et al., 2015). A previous study also found the antagonistic regulation between H3K36me3 and gene expression level (Yang et al., 2014). A lack of H3K36me3 resulted in a fully silenced state at *FLC* gene (Yang et al., 2014). In this study, we analyzed the association among anti-sense RNA, sense RNA, and H3K36me3 modification. We found that anti-sense RNA was negatively correlated with its cognate sense RNA, which was associated with the decreasing enrichment of H3K36me3 modification (Figure 5). This result suggested that anti-sense RNA inhibited sense RNA expression by decreasing H3K36me3 modification. It was also the first study to explore the regulated relationship between epigenetics modification with the expression of anti-sense and sense RNA by analyzing large-scale sequencing data.

## Conclusion

Our study was the first to analyze the expression complementation of anti-sense transcripts in two maize inbred lines and their F_1_ hybrid. A total of 293 and 242 SPEA transcripts were detected in immature ear and leaves (Figure 3). Negative expression correlation was found between SPEA transcripts and their cognate sense transcripts (Figure 4). Interestingly, we found that anti-sense transcripts inhibited their sense transcripts by reducing enrichment of H3K36me3 modification (Figures 4, 5). This study demonstrates the value of studying the mechanism of transcriptome complementation in maize by integrating epigenetic information.

## Materials and Methods

### Plant Materials

Three maize genotypes (B73, Mo17, and their F_1_ hybrid) were obtained from Prof Lai lab and planted in Guangzhou, China, on March 2019. The immature ear (ear length about 3–4 cm) and 14-day-old leaves were sampled with three biological replicates. A total of 18 samples were immediately frozen in liquid nitrogen and then stored at into −80°C prior to use in the experiments.

### Library Construction and Sequencing

Total RNA was extracted from immature ear and leaves using TRIzol (Invitrogen, United States) and treated with RNase-free DNase I. RNA integrity was determined by > 8 on a 2100 Bioanalyzer (Agilent Technologies). Libraries for strand-specific RNA-seq were constructed as described (Hirsch et al., 2014). Briefly, mRNA was purified by OligoTex mRNA Midi Kit (Qiagen) and mixed with RNA fragmentation reagents (Ambion). The first-strand cDNA was synthesized using random hexamer primers, and second-strand cDNA was synthesized with dUTP. Double-stranded cDNA fragments were purified and then ligated with adaptors. Second-strand cDNA was removed by digesting dUTP with AmpErase UNG (Applied Biosystems). Libraries were sequenced at Biomarker Technologies Corporation (Beijing, China) using Illumina deep sequencing, following the manufacturer’s instructions.

### Processing Strand-Specific RNA-seq Data

Sense and anti-sense transcripts were identified with the following several steps. (1) Filtering reads: Raw reads were processed with FASTX Toolkit^1^. Low-quality and adaptor sequence reads were removed. (2) Mapping reads: The filtered reads were aligned to maize reference genome (B73 maize Version 4) (Jiao et al., 2017) by hisat2 software with default parameters (Kim et al., 2015). (3) Assembling anti-sense transcripts: BEDTools software (v2.25.0) (Quinlan and Hall, 2010) were applied to split reads into minus and plus strands. Reads with the strand direction opposite that of annotated transcripts for each gene were used to assemble anti-sense transcript by StringTie software (version 1.2.3) (Pertea et al., 2015) with a required minimum junction coverage of 5 (-j) and 5 minimum reads per bp coverage to be considered for transcript assembly (-c). (4) Defying sense and anti-sense transcript pair: With the result of step 3, sense transcript was defined as the longest transcript based on annotated genes, and its cognate anti-sense transcript was overlapped with > 50 bp. NAT pairs located at overlapped genes that transcript from opposite strands were filtered. In this way, one sense transcript might contain more than one anti-sense transcript. (5) Quantifying expression level of sense and anti-sense transcript: The read counts from each transcript (sense and anti-sense transcript) were normalized to FPKM value were used to quantify transcript expression level by cufflinks with default parameters (Trapnell et al., 2012). The ratio of reads between sense and anti-sense transcript was between 0.01 and 100.

### Principal Component Analysis

The package factoextra in R language was applied to perform PCA.

### H3K36me3 Analysis

The public H3K36me3 data were obtained, which were generated by next-generation sequencing in leaf tissue (14 days old) of three inbreed lines, including B73, Mo17, and their hybrid (Murray et al., 2015). The sequencing data were mapped to maize reference genome (B73 Version 4) by bowtie2 software with default parameter (He et al., 2013). The H3K36me3 enrichment level of genes was calculated with the following formula: Level(gene) = Reads (gene)/Length(gene)/Depth(sample)/10^9^, where Reads (gene) is the mapped reads from upstream 500 bp to downstream 500 bp of gene, Length(gene) is the length of gene, and Depth(sample) is the total sequencing reads for a given sample.

### Experimental Validation of Sense and Anti-sense Transcripts

Strand-specific qPCR was performed to quantify sense and anti-sense transcripts for inbred lines B73 and Mo17 in immature ear and leaf tissues. The total RNA used for qPCR was the same RNAs used for library construction. Total RNA was treated with 2 U DNase I (Takara) for 30 min at 37°C and then was incubated at 85°C for 10 min. First-strand cDNA synthesis was conducted using primers (Supplementary Table 3). The protocol for strand-specific amplification has been described in detail (Ho et al., 2010). Specific primers of each pair of sense and anti-sense transcript were used for qPCR analysis, and 18s rRNA and glyceraldehyde-3-phosphate dehydrogenase (GAPDH) were amplified as endogenous housekeeping controls. Relative gene expression for a given gene in a sample was determined based on the cycle threshold (CT) value of target gene and housekeeping gene using the following formula: 2^–ΔCT^ (ΔCT = CT_target_ - CT_housekeeping_). For each sample, qPCR was conducted with three technical triplicates. Strand-specific PCRs were initially denatured at 94°C for 5 min and then amplified over 35 cycles of denaturation at 94°C for 1.5 min, annealing at primer-specific temperatures for 1 min, and extension at 72°C for 2 min. Reactions were held at 4°C, and PCR products were visualized on 1% agarose. qPCR was performed on the Bio-Rad CFX96 real-time PCR System (Bio-Rad, CA) under the cycling conditions (95°C for 1 min, 40 cycles of 95°C for 5 s, and primer-specific temperatures for 30 s). The melting curves were generated at 60–95°C after 40 cycles to check for primer specificity.

### Statistical Approach

The multiple comparison tests in Figures 1, 5 were done by function “duncan.test” in R language. PCC and *P*-value to calculate the correlation between sense and anti-sense transcript (Supplementary Figure 4) were done by function “cor.test” in R language.

## Data Availability Statement

The datasets GENERATED for this study can be found CNBC-NGDC (https://bigd.big.ac.cn), accession CRA003636.

## Author Contributions

YQ and XBZ designed the research. XZ analyzed the data, wrote the manuscript, and carried out the experiments. Both authors read and approved the manuscript.

## Conflict of Interest

The authors declare that the research was conducted in the absence of any commercial or financial relationships that could be construed as a potential conflict of interest.

## Footnotes

1 http://hannonlab.cshl.edu/fastxtoolkit/

## Supplementary Material

Supplementary Figure 1 PCC value between replicates using sense RNA expression.

Supplementary Figure 2 PCC value between replicates using anti-sense RNA expression.

Supplementary Figure 3 Expression heatmap of SPEA and its cognate sense RNA identified in immature ear tissue in three genotypes (B73, Mo17, and BM). Expression profile of SPEA_B (anti-sense) **(A)** and its cognate sense transcript **(B)** in three genotypes; Expression profile of SPEA_M (anti-sense) **(C)** and its cognate sense transcript **(D)** in three genotypes.

Supplementary Figure 4 Expression correlation ship between anti-sense and sense transcripts. **(A)** For SPEA_B detected in leaf tissue, the relative fold change of anti-sense (B73/Mo17) and sense transcript (B73/Mo17) was plotted. **(B)** For SPEA_M detected in leaf tissue, the relative fold change of anti-sense (B73/Mo17) and sense transcript (B73/Mo17) was plotted. The *r*^2^ (PCC) was calculated by the fold change value of anti-sense and sense transcript.

Supplementary Figure 5 qPCR validation of sense and anti-sense transcripts using four genes.

Supplementary Table 1 Summary of sequencing data.

Supplementary Table 2 SPEA list identified in immature ear and leave tissues.

Supplementary Table 3 Primer used for experimental validation.
