# Supplementary figures and images for: Single-Parent Expression of Anti-sense RNA Contributes to Transcriptome Complementation in Maize Hybrid
Source: Front Plant Sci. 2020 Dec 3;11:577274. doi: 10.3389/fpls.2020.577274 (PMC7744309; doi:10.3389/fpls.2020.577274)

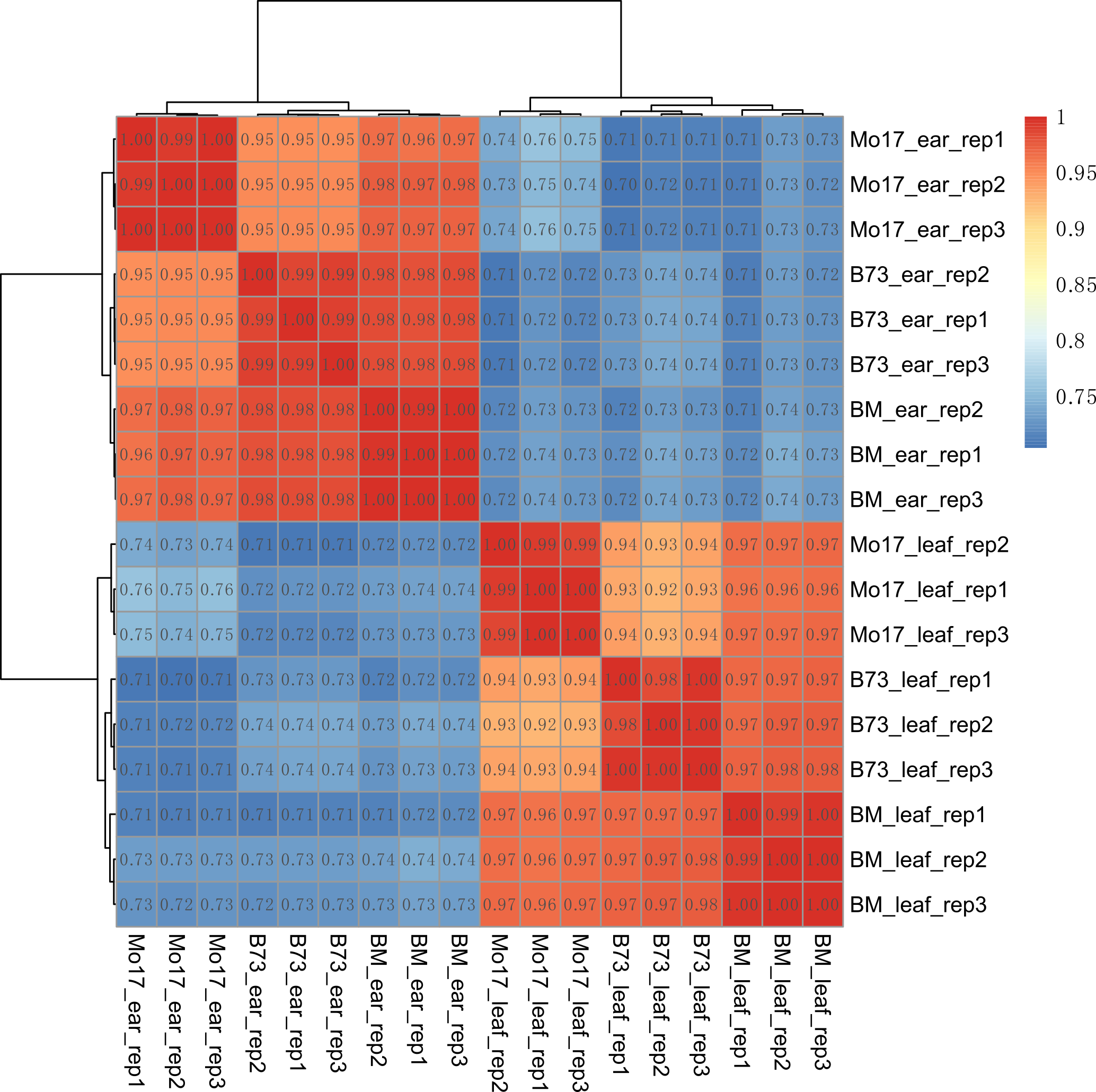

Supplement: Supplementary Figure 1 — PCC value between replicates using sense RNA expression. [file Image_1.TIF]

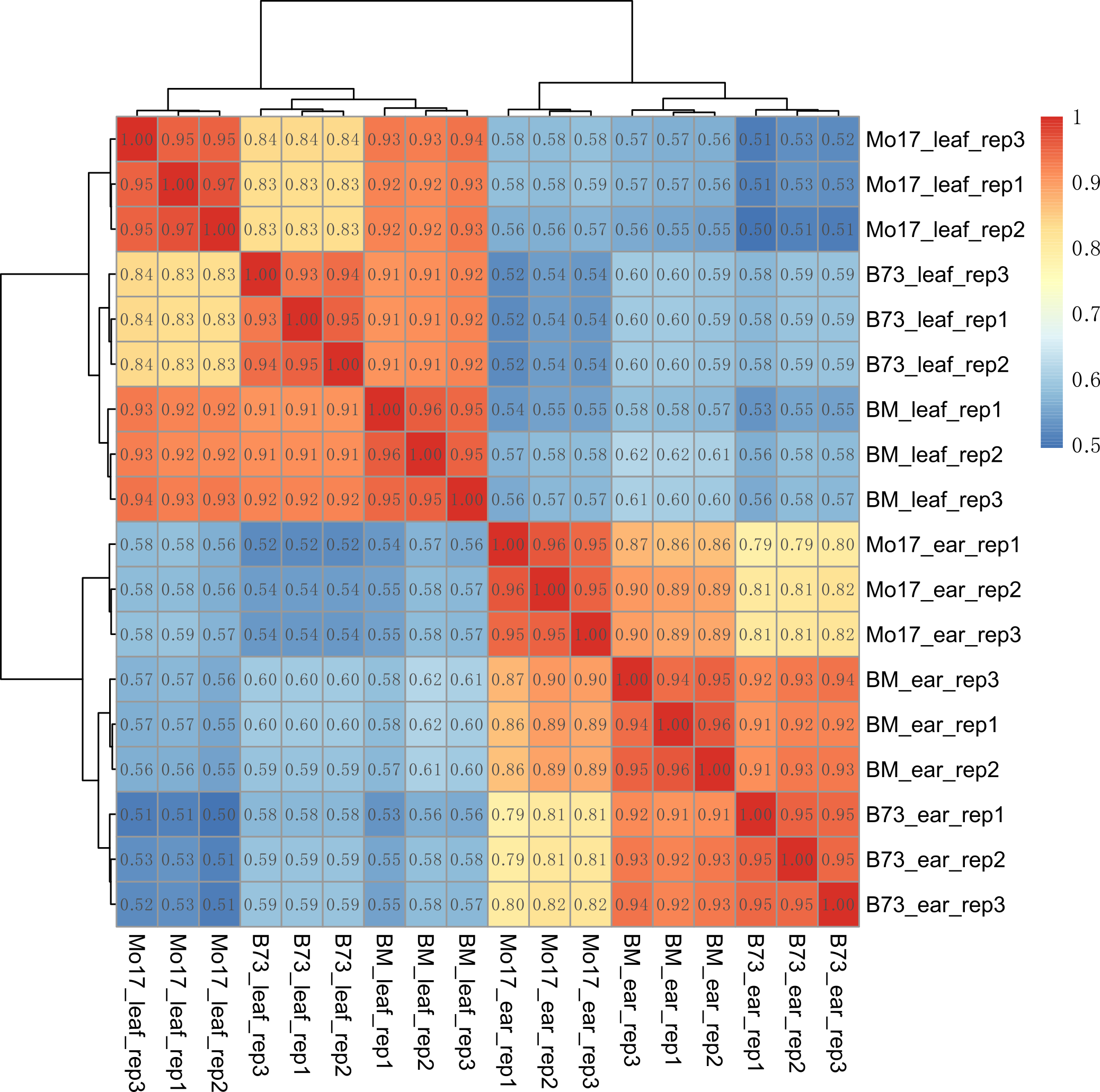

Supplement: Supplementary Figure 2 — PCC value between replicates using anti-sense RNA expression. [file Image_2.TIF]

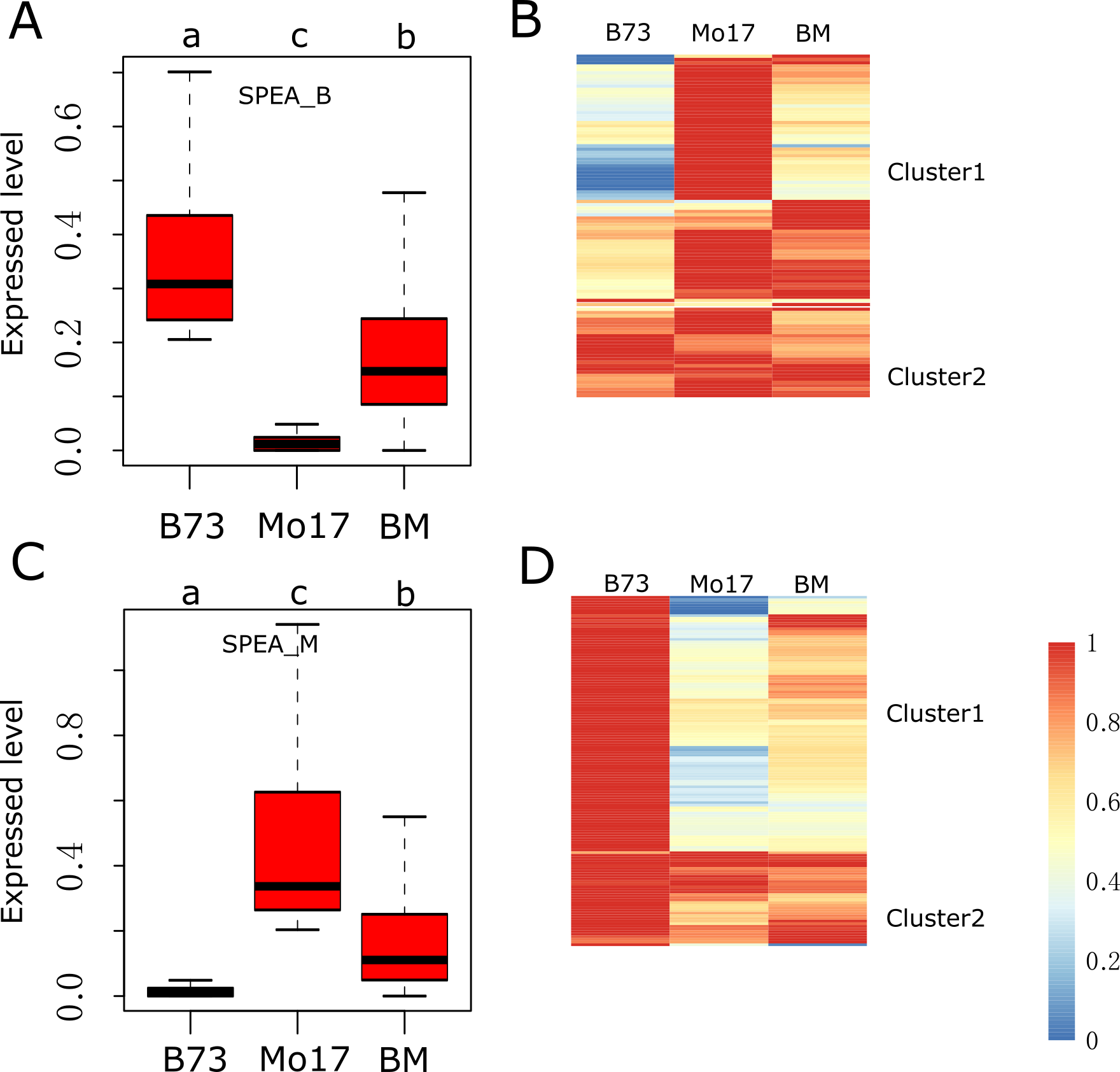

Supplement: Supplementary Figure 3 — Expression heatmap of SPEA and its cognate sense RNA identified in immature ear tissue in three genotypes (B73, Mo17, and BM). Expression profile of SPEA_B (anti-sense) (A) and its cognate sense transcript (B) in three genotypes; Expression profile of SPEA_M (anti-sense) (C) and its cognate sense transcript (D) in three genotypes. [file Image_3.TIF]

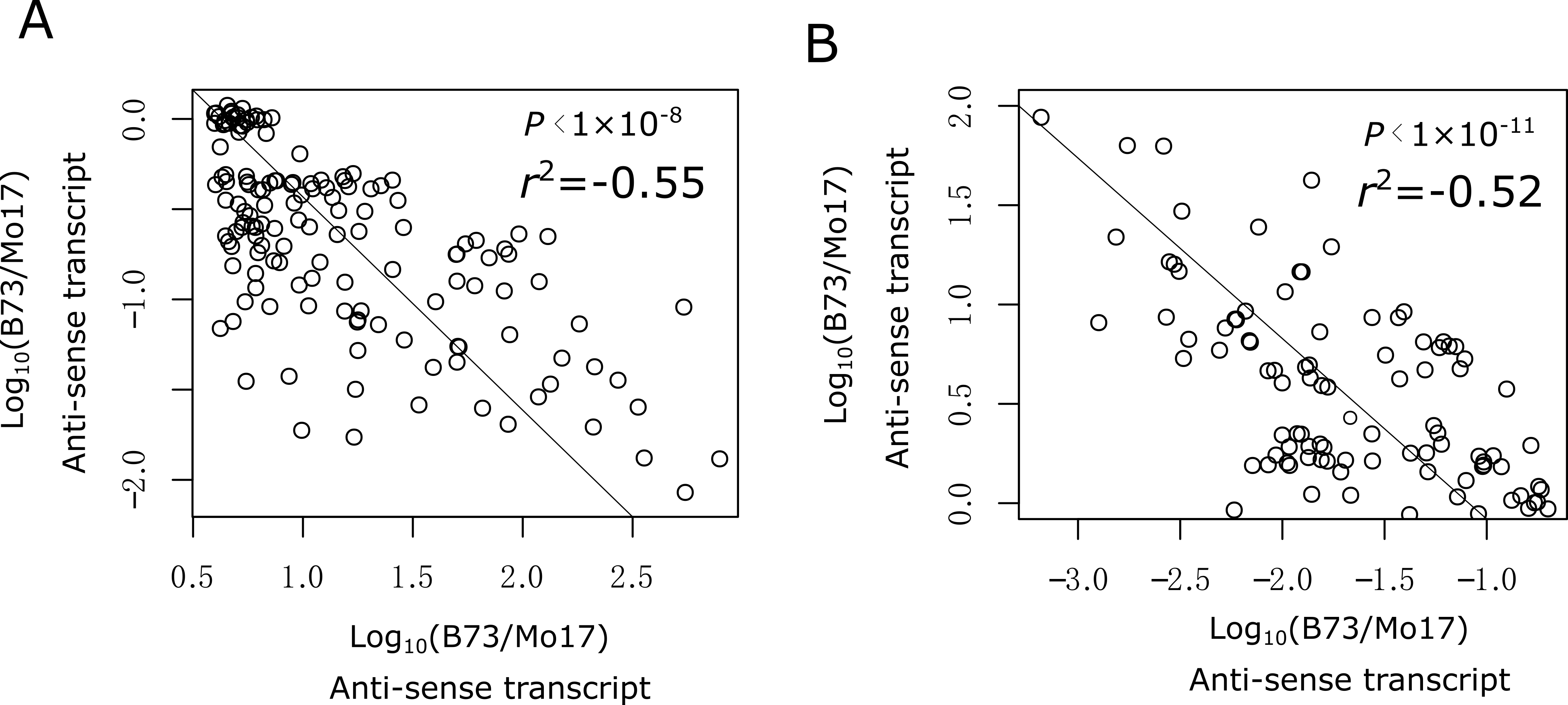

Supplement: Supplementary Figure 4 — Expression correlation ship between anti-sense and sense transcripts. (A) For SPEA_B detected in leaf tissue, the relative fold change of anti-sense (B73/Mo17) and sense transcript (B73/Mo17) was plotted. (B) For SPEA_M detected in leaf tissue, the relative fold change of anti-sense (B73/Mo17) and sense transcript (B73/Mo17) was plotted. The r2 (PCC) was calculated by the fold change value of anti-sense and sense transcript. [file Image_4.TIF]

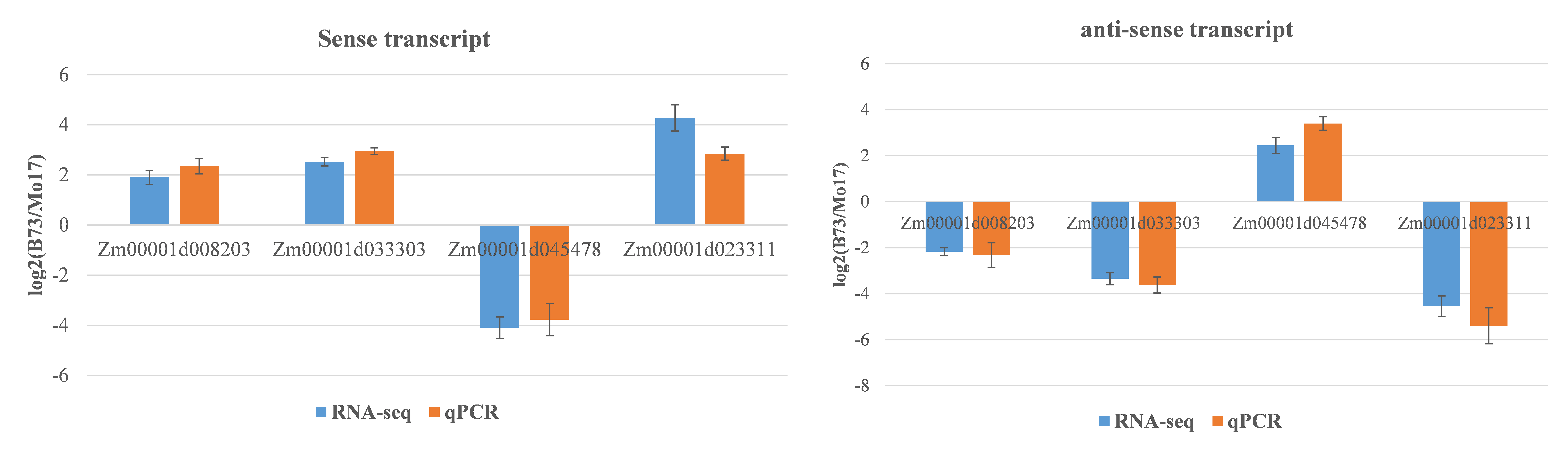

Supplement: Supplementary Figure 5 — qPCR validation of sense and anti-sense transcripts using four genes. [file Image_5.TIF]
